# Structure of the Chemical and Genetic Diversity of the True Lavender over Its Natural Range

**DOI:** 10.3390/plants9121640

**Published:** 2020-11-24

**Authors:** Yolande Despinasse, Sandrine Moja, Catherine Soler, Frédéric Jullien, Bernard Pasquier, Jean-Marie Bessière, Camille Noûs, Sylvie Baudino, Florence Nicolè

**Affiliations:** 1 Université de Lyon, UJM-Saint-Etienne, CNRS, LBVpam FRE 3727, 23 rue du Dr Paul Michelon, F-42023 Saint-Etienne, France; yolande.despinasse@gmail.com (Y.D.); sandrine.moja@univ-st-etienne.fr (S.M.); c_soler@orange.fr (C.S.); jullien@univ-st-etienne.fr (F.J.); sylvie.baudino@univ-st-etienne.fr (S.B.); 2 Conservatoire National des Plantes Médicinales Aromatiques et Industrielles, Route de Nemours, 91490 Milly La Forêt, France; bernard.pasquier@cnpmai.net; 3 Ecole d’Enseignement Supérieur en Chimie de Montpellier, 8 Rue de l’École Normale, 34090 Montpellier, France; jeanmarie.bessiere@gmail.com; 4 Laboratoire Cogitamus, 1 ¾ rue Descartes, 75005 Paris, France; camille.nous@cogitamus.fr

**Keywords:** *Lavandula angustifolia* Miller, sp. *pyrenaica*, *Lavandula austroapennina*, DAPC, chemotype, terpene

## Abstract

The true lavender *Lavandula angustifolia* Miller is a Mediterranean aromatic shrub widely cultivated for its high quality essential oil used in perfumery and phytotherapy. Despite its economic importance, the intra-specific diversity among wild, non-cultivated plants remains poorly understood. We analyzed the structure of the chemical and genetic diversity of plants from 14 sites sampled over the entire native range of the true lavender. Volatile organic compounds of inflorescences were analyzed using gas chromatography coupled to mass spectrometry. Genotyping was performed with fingerprinting genetic markers. To limit the influence of environmental variability on chemical composition, plants were grown in the same conditions in a common garden. Without prior knowledge, discriminant analysis of principal component identified unambiguously four distinct chemotypes among three genetic populations. Co-inertia analysis and supervised analysis which integrated multiple datasets indicated a strong congruency between chemical and genetic patterns. Two distinct genetic units were located at the edge of the distribution area in the south of Italy and in the northeast of Spain, and were associated with two distinct chemotypes. Our results confirmed the existence of three genetically distinct entities, suggesting speciation. All French populations and the Italian Piedmontese population were genetically homogeneous but separated in two distinct chemotypes. The dominant chemotype was present in the center of the native range in southeastern France and was at the origin of the current most cultivated French varieties. Its main compounds were linalyl acetate, linalool, and caryophyllene oxide. The second French chemotype was found in south of Massif Central and presented high abundance of valuable linalyl and lavandulyl acetates. Linalool, eucalyptol, β-caryophyllene, borneol, camphor, and cis-sabinene-hydrate were significantly associated with southern latitudes and their role would be worth exploring.

## 1. Introduction

The true lavender (*Lavandula angustifolia* Miller) is a medicinal and aromatic plant of the Lamiaceae family. The plant grows in an open arid habitat and is endemic to the Mediterranean climate. The distribution of the species is determined by the occurrence of calcareous sedimentary rocks and schists. The natural range includes the south of France, the French and Spanish Pyrenees, and the Italian Alps [1]. Based on morphology, Upson and Andrews [1] identified two subspecies: *L. angustifolia* sp. *angustifolia* in southern France and northern Italy, and *L. angustifolia* sp. *pyrenaica* in the Pyrenees. The Pyrenean lavender is characterized by broader bracts, larger flowers with a darker hue, and a felt-like indumentum of the calyx due to short highly branched hairs. More recently, based on morphological and phytochemical data, Passalacqua et al. [2] described a new species “austroapennina” of lavender in an isolated location of true lavender in the south of Italy and proposed to raise the subspecies *pyrenaica* to species level according to the phylogenetic study of Moja et al. [3]. The study of the structure of genetic diversity of these accessions, compared to the other wild populations of true lavender, could provide insights on their status of species or population.

The subspecies *angustifolia* represents one of the most economically important taxa in the genus, due to the properties of its essential oil (EO) used in perfumery, cosmetics, and therapeutics since antiquity [4]. In Provence and Bulgaria, *L. angustifolia* sp. *angustifolia* was domesticated in the early twentieth century [5]. Seeds from wild populations were selected for yield and EO quality, and grown in fields. In the 1950s, to meet the increasing demand on lavender EO and standardize yield and composition, cultivars were created by developing softwood and hardwood cutting techniques. The development of the culture of lavender worldwide led to the definition of international standards to assess the quality of the EO (ISO 3515:2002/Cor 1:2004, https://www.iso.org and NF ISO 3515:2004 (T75-301), https://www.boutique.afnor.org). Lavender EO is rich in mono- and sesquiterpenes that are volatile organic compounds (VOCs) [1,6]. The two major compounds are monoterpenes: linalyl acetate (from 25 to 45%) and linalool (from 22 to 38%). Together with lavandulol (more than 0.3%) and lavandulyl acetate (more than 2%), these compounds are the most valuable components for high quality EO according to the French and international standards for lavender EO because they have a “floral” scent characteristic of lavender EO ([7], Table 1). On the contrary, the quality of EO is depreciated if camphor, limonene, and β-phellandrene exceed 0.5% each, eucalyptol and α-terpineol exceed 1%, and octanone-3 exceeds 2%. These compounds have spicy, strong, lemony, and/or pungent scent while the ISO standard states that the EO of lavender must have a “fresh, floral smell, recalling that of the inflorescence of the plant” (NF ISO 3515:2004 (T75-301), https://www.boutique.afnor.org).

With the increasing demand for natural products, the essential oil industry is flourishing and presents a projected global market value of $7.47 billion USD in 2018. *Lavandula* essential oils contribute largely to this growing industry with approximately 1500 tons produced annually [8]. Despite the economic importance of lavender EO, varietal creation programs select cultivars without any background on the genetic and chemical diversity of the wild populations from which they originate. Indeed, to date, no inventory has been conducted in the wild populations over the natural range of the true lavender. However, the French lavender industry, the world’s 2nd largest producer of lavender essential oil, is facing the emergence of disease, supporting the need to diversify cultivars [9]. Surprisingly, the study of chemical diversity was conducted for non-cultivated or marginally cultivated species of the genus *Lavandula* such as *L. stoechas* and *L. multifida*. In *L. stoechas*, different studies identify a high level of variability in EO composition within wild populations collected in different areas (Crete and Greece [10]; Algeria [11]). In *L. multifida*, the three chemical groups identified within 12 wild Tunisian populations do not show a clear pattern with bioclimatic or geographical regions [12]. On the contrary, in the Spike lavender *L. latifolia*, the chemical profiles are variable amongst geographical regions and are correlated with bioclimatic belts [13]. Populations with high proportions of linalool are associated with supra-Mediterranean climate, while the thermo-Mediterranean climate promotes eucalyptol accumulation and low proportion of linalool. However, because plants were collected directly from the field in this study, it is impossible to disentangle the effect of the environment (phenotypic plasticity) and the effect of genotypes (local adaptation) to explain the variation in secondary metabolites. Phenotypic plasticity is a rapid and reversible phenotypic change in individuals exposed to environmental change. In opposition, local adaptation is the result of natural selection and implies the transmission of heritable characters. Depending on the environment, certain traits will allow a better survival and/or reproduction of individuals and the genes that determine these traits will be selected from one generation to the next. For example, isoprene was demonstrated to help tobacco and poplar recovering from high temperature exposure [14,15] or deter lepidopteran caterpillars of *Manduca sexta* from feeding in transgenic isoprene-emitting tobacco [16].

Genetic inheritance of different chemical signatures within a species introduces the concept of chemotype, which is a chemically distinct entity within the same species. In *Thymus vulgaris*, six chemotypes (carvacrol, thymol, linalool, thuyanol-4, α-terpineol, and geraniol) were identified and related to allelic variants using crossing experiments [17]. These six chemotypes show clear spatial segregation because two phenolic chemotypes (carvacrol and thymol) are freezing sensitive while linalool, thuyanol-4, α-terpineol, and geraniol chemotypes are freezing tolerant [18]. High intra-specific diversity is the basis of adaptation and allows plants to evolve rapidly. While plant ontogeny, environmental and genetic variation are recognized as sources of chemical variation, efforts should be made to understand the relative contributions of genetic and environmental variation on chemical variation [19]. Inherited characters associated with adaptive traits should be considered to improve varietal selection and select locally well-adapted cultivars.

In this study, we analyzed the structure of chemical and genetic diversity of plants grown from 14 wild locations of true lavender over its native range, including Spanish and south Italian sites at the edge of the range. To disentangle phenotypic plasticity from local adaptation, seeds were collected in the wild and were grown in the same conditions in a common garden. Samples were collected at the same date on plants of the same age to limit the effect of ontogeny and weather conditions. For comparison with domesticated lavender, four different cultivars amongst the most cultivated in France (Matheronne, Maillette, Diva, and Rapido) were included in the study. Plants were genotyped using amplified fragment length polymorphism (AFLP) fingerprinting markers and the content of terpenes in the inflorescences was obtained using gas-chromatography coupled with mass spectrometry. We inferred independently the structure of the chemical and genetic diversity using an unsupervised discriminant approach based on principal components, called DAPC (discriminant analysis on principal component [20]). This method was chosen because it integrates the two steps of analysis: identify the optimal number of groups (unsupervised analysis) and pinpoint discriminant variables between these identified groups (supervised analysis). The congruency between the patterns of variation of chemical and genetic data was analyzed with a multidimensional co-inertia analysis and a novel supervised analysis which integrated multiple datasets. Finally, major and discriminant compounds were correlated with latitude and altitude of the sampled populations.

## 2. Results

### 2.1. Global Composition and Amount of VOCs

We identified a total of 63 repeatable VOCs from our entire data set (14 sampled sites × 4.6 replicate plants per site + 25 plants from cultivated fields), which varied per site in the mean between 10.7 (Fam) and 55.2 (Ica) Table S1, Table 2). The 63 repeatable compounds represent more than 70% of the total abundance for 12 out of 14 populations. The remaining 30% of the total peak area of the chemical profile corresponds to poorly repeatable peaks, co-eluted peaks, pollutants, or artifacts. In the two remaining populations, Fam and Ica, the identified compounds represent 59% and 67% of the total area, respectively. In Fam, only small amounts of VOCs were detected and peaks were not well defined. In Ica, plants were particularly diverse in terpenes and several small peaks were not included in the pool of 63 repeatable compounds. Overall, in the sampled sites, the bouquet of VOCs is mainly composed of monoterpenes (ranging from 48 to 86% of the total amount, Table 2) and secondarily sesquiterpenes (ranging from 4 to 17%, Table 2). Other compounds, such as 3-octanol, 3-octanone, octen-3-ol, represent only 2.6% of the total on average (Table S1).

### 2.2. Unsupervised Identification of the Structure of Individuals

**Figure 1:**
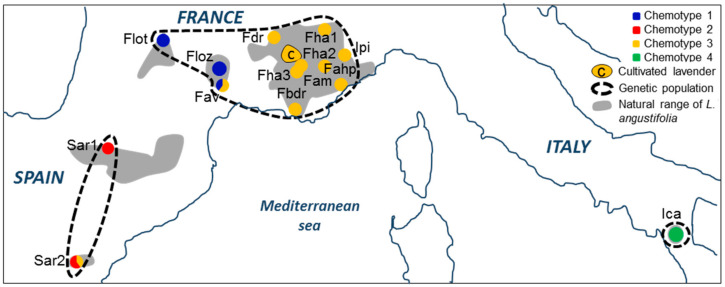
Chemical and genetic structure of the 14 native sampled sites (N = 65) and 5 cultivated fields (N = 25). Grey shading indicates the natural range of the true lavender *Lavandula angustifolia*. The position of the pie chart indicates the original location of the sampled site. The color of the pie chart designates the chemical cluster membership. The black dashed lines delimit the three genetic units identified with DAPC (discriminant analysis on principal component) on the AFLP (amplified fragment length polymorphism) fingerprinting genotypes. The cultivated fields are grouped in an area with the letter c.

We first ran the unsupervised clustering method of DAPC on the chemical composition of sampled plants. We retained 20 principal components with 98% of inertia. The optimal number of clusters that maximized the inter-group variance and minimized the BIC was 4. We used the optimization and validation procedures (*optim.a.score* and *xvalDapc*) to determine the optimal parameters to construct the discriminant model. We ran the final discriminant analysis on the 4 first PCs (76% of inertia) to assign the individuals to the four groups. Stratified cross-validation with 1000 repetitions gave 99.2% of good assignment with two discriminant axes. The Figure 1 clearly highlights a correlation between cluster membership of the individuals and geographic location.

**Figure 2:**
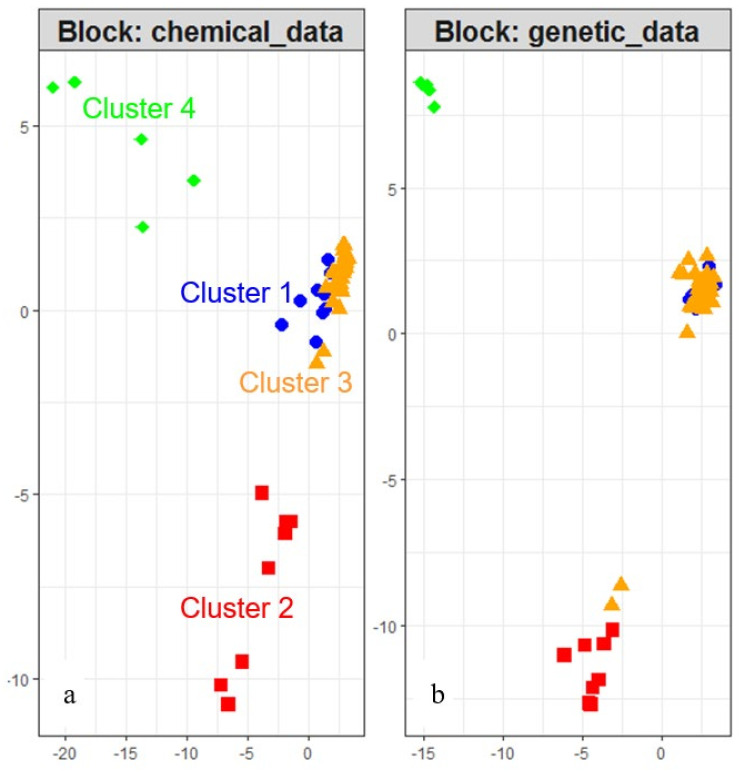
Congruency of the structures of individuals between chemical (**a**) and genetic dataset (**b**), resulting from DIABLO (data integration analysis for biomarker discovery using latent variable approaches for omics studies) analysis. Each point represents one individual placed according to its projection in the subspace defined by the latent variables of the multivariate model (N_total_ = 90; N_cluster1_ = 12; N_cluster2_ = 8; N_cluster3_ = 65; N_cluster4_ = 5). This plot enables to visualize similarities between samples. The four different symbols materialized the 4 chemotypes identified by DAPC on 90 individuals of 14 wild sampled sites of lavender and 5 cultivated fields.

Cluster 4 is composed of the 5 samples originating from the isolated Calabrese area (Ica), at the southeastern limit of the species range. Cluster 2 corresponds to the individuals of the Spanish areas (all individuals of Sar1 and 3/5 individuals of Sar2). In France, two distinct chemical profiles are found. Cluster 1 groups individuals from the south of Massif Central (all plants from Flot and Floz and 2/5 of Fav), while cluster 3 covers all samples located in the center of lavender native range (Fdr, Fha1, Fha2, Fha3, Fbdr, Fam, Fahp, Ipi) together with all cultivars. Two individuals from the Spanish site Sar2 and three individuals from the south of Massif Central (3/5 Fav) are also assigned to the cluster 3 (subsequently named Provencal cluster). In a second step, we run DAPC on the genetic data. The optimal number of genetically distinct groups is unambiguously evaluated to 3. Indeed, the cross validation process finds a success of assignment of 100%, whatever the number of PCs selected. One group corresponds to the isolated Calabrese plants (Ica), another contains the Pyrenean plants (all individuals of Sar1 and Sar2) and the last group clusters all French individuals and the Italian Piedmontese plants (Figure 1). The procrustean co-inertia analysis (PCIA) measures the congruency between the structure of wild individuals from chemical and genetic data. The m^2^ is high (0.60) and significant (*p* = 0.001, 999 permutations), indicating a good concordance between the two datasets. The DIABLO analysis is a complementary approach to co-inertia analysis (DIABLO: Data Integration Analysis for Biomarker discovery using Latent variable approaches for Omics studies, [21]). It combines the two datasets to better understand the interplay between all variables. The Figure 2 materializes the three genetic populations on the right and confirms graphically the congruence of the structures of the individuals at the chemical and genetic level.

Interestingly, the two French chemotypes belong to the same genetic population. The two individuals of Sar2 presenting the chemical composition of the cluster 3 (two triangles among the squares in Figure 2b) are genetically similar to other Spanish plants. The genetic groups are more homogeneous than the chemical clusters. The analysis of molecular variance AMOVA indicates that most of the molecular variance is among genetic clusters (53%), 15% is among sampled sites within genetic clusters, and 32% within sampled sites (Table 3). The pairwise PHI_PT_ between genetic clusters indicates a high level of differentiation with PHI_PT_ ranging from 0.552, between French and Spanish clusters, to 0.752 between Calabrese and Spanish clusters (Table 4).

### 2.3. Composition of the Four Lavender Chemotypes

**Figure 3:**
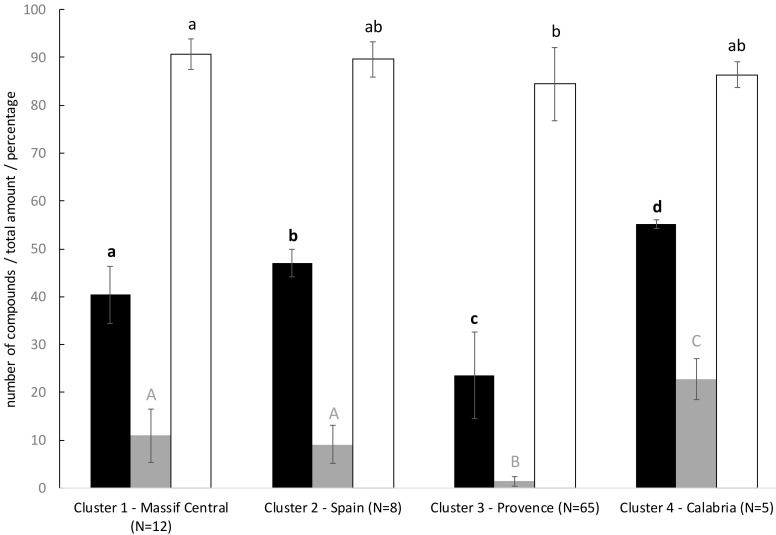
Number of compounds (black bars), total amount of volatile compounds (grey bars; internal standard equivalent per g of dry inflorescence) and percentage of monoterpenes (white bars) in the four clusters identified by DAPC on the chemical data. Black bold letters in the lower case indicate significant difference in the number of compounds between clusters. Grey letters in the upper case indicate significant difference for total amount of volatile compounds. Black letters in the lower case indicate significant difference for percentage of monoterpenes. Pairwise comparisons were performed with pairwise Wilcoxon tests. The significance level was set to *p* = 0.05.

The four different chemotypes differ by their total amount of VOCs, their number of compounds, and their composition (Figure 3). The Calabrese cluster 4 has the highest significant number of compounds (55.2 vs. 47.0 for cluster 2, 40.9 for cluster 1, 23.4 for cluster 3, Kruskal–Wallis chi-squared = 41.5, df = 3, *p* < 0.001) and the highest total amount (35.2 vs. 12.1 for cluster 1, 10.4 for cluster 2, and 1.7 for cluster 3, Kruskal–Wallis chi-squared = 44.1, df = 3, *p* < 0.001). On the contrary, the Provencal chemotype (cluster 3) is the poorest. The mean percentage of monoterpenes ranges from 84.5 (cluster 3) to 90.7 (cluster 1) and only differs significantly between the two French chemotypes (Figure 3).

**Figure 4:**
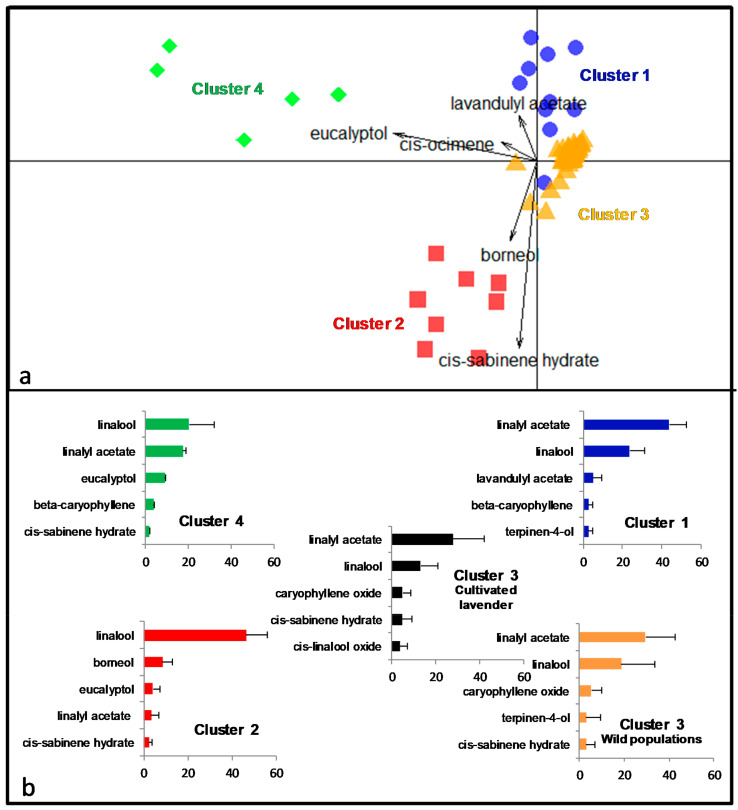
(**a**) Discriminant analysis based on the chemical composition of 90 individuals from 14 wild sampled sites and 5 cultivated fields of lavender (N_cluster1_ = 12; N_cluster2_ = 8; N_cluster3_ = 65; N_cluster4_ = 5). The arrows indicate the most discriminant compounds. The different symbols materialized the 4 clusters identified by DAPC. (**b**) For each cluster, the mean percentage of the total amount ± SD for the five major compounds is represented. For cluster 3, the composition of wild (N = 40) and cultivated (N = 25) populations are separated.

The redundancy analysis RDA detected significant differences in chemical composition between clusters (F = 40.6, df = 3.58, *p* < 0.001). A pairwise post-hoc comparison indicates that the chemical composition of all clusters differs significantly from each other (*function pairwise.factorfit*, all pairwise *p*-value < 0.01). Figure 4a shows a graphical representation of the first two axes of the discriminant model (99.2% of inter-cluster variance). The first axis separates the Calabrese cluster 4 and the Spanish cluster 2 from the two French clusters, while the second axis disentangles the two French clusters and separates the cluster 2.

As commonly observed in true lavender, the cultivated lavender is classically dominated by linalyl acetate and linalool, a common pattern shared by the French cluster 1 and 3 (Figure 4b). In cluster 4, the order is reversed with linalool as the major compound. The Spanish cluster 2 shows a clear distinct chemical profile dominated by linalool and borneol. Linalyl acetate is not in its five major compounds. Five main discriminant compounds are identified according to their contribution on the two first discriminant axis. Eucalyptol is characteristic of the Calabrese and Spanish cluster where it is the third major compound. Borneol and cis-sabinene hydrate are associated with the Spanish cluster 2. The south of Massif Central cluster differs from the other clusters by its higher amount of the valuable lavandulyl acetate. Cis-ocimene is characteristic of cluster 1 and 2, representing 2.55 and 1.89% of the total respectively while the other clusters have lower value (0.19 and 0.45%). Interestingly, the Provencal cluster 3, with 41 samples, presents the highest number of samples but the most homogeneous chemotype. It has the lowest intra cluster variability with yellow triangles tightly grouped in Figure 4a.

Interestingly, when correlating the latitude and altitude of the original wild populations with discriminating and major compounds, negative and significant correlations are found between latitude and linalool (Spearman rho = −0.30, *p* = 0.016), eucalyptol (Spearman rho = −0.50, *p* < 0.001), β-caryophyllene (Spearman rho = −0.39, *p* = 0.002), borneol (Spearman rho = −0.55, *p* < 0.001), camphor (Spearman rho = −0.49, *p* < 0.001), and cis-sabinene hydrate (Spearman rho = −0.34, *p* = 0.007). These negative correlations may indicate a higher abundance of these compounds in southern populations. Terpinen-4-ol is positively correlated with latitude (Spearman rho = 0.30, *p* = 0.02) and negatively correlated with altitude (Spearman rho = −0.39, *p* = 0.002). Camphor is positively correlated with altitude (Spearman rho = 0.30, *p* = 0.02) but the correlation is essentially driven by the high amount of camphor in the two Spanish populations located at 1000 and 1450 m (Table 1). 

## 3. Discussion

This study is the first to analyze the structure of the chemical and genetic diversity of natural populations of true lavender, over its native range. The composition in secondary metabolites of the inflorescences was determined for 65 samples originating from 14 natural sampled sites of the true lavender. The same plants were genotyped with 206 repeatable AFLP markers. Twenty-five samples from 4 cultivars within 5 cultivated fields were included in the study to establish the link between wild populations and cultivated varieties. Genetic and chemical data were used to infer population structure by determining homogeneous groups without prior knowledge. The discriminant analysis of principal components (DAPC, [22]) was successfully performed on both genetic and chemical data to infer the optimal number of clusters among the individuals without labeling the data (no information about geographical localization and population membership). DAPC is a multivariate method that uses a prior ordination step with principal component analysis (PCA). First principal components are defined as the new synthetic variables to construct a discriminant analysis (DA). This approach ensures that variables submitted to DA are perfectly uncorrelated, normally distributed, and that their number is less than the number of samples. Thus, it overcomes the problems of multicolinearity, overfitting, and non-normality usually associated with chemical data. We preferred DAPC to classical clustering approaches such as hierarchical clustering or classical k-means because it assists in choosing the optimal parameters (optimization and validation) and allows evaluating the clustering with inter-group variance and Bayesian information criterion (BIC). For the discriminant part of the analysis, DAPC and partial least square discriminant analysis were both implemented and gave very similar results but we chose DAPC because results were more robust in terms of cluster membership to changes in model parameters. Finally, DAPC identified unambiguously three distinct genetic units and four distinct chemotypes among the natural populations of true lavender (Figure 1).

Methodological advances in statistical approaches facilitate multi-dataset analysis. When data of different natures are collected on the same sample, two different approaches are available and complementary: co-analysis and combined integrative analysis. Co-analysis uses classically procrustean co-inertia analysis and aims at prospecting for the common structure of the data. Each dataset is ordinated separately and co-inertia superimpose the two ordinations in a second time to identify common pattern. On the other way, integrative analysis combines the different datasets in one file before the analysis. It aims at identifying variables that are highly correlated between the datasets and discriminative between *a priori* known groups of samples (see [23] for details about use of these methods, scripts, and interpretation of the results). In our study of chemical and genetic diversity of true lavender, the two different methods give highly congruent results, providing confidence in the biological message. Two genetically homogeneous populations, the Calabrese population and the Spanish populations, express their own differentiated chemotype, while the third genetic population presents two distinct chemotypes.

One of the three genetic units is composed of the two Spanish sites. Eight of 10 plants of these sampled sites form the Spanish chemotype. When seeds were collected on site in 1998 and 1999, the botanist had classified the plants, based on morphological traits, in the subspecies *Lavandula angustifolia* sp. *pyrenaica*. Our results showing the high genetic differentiation of this cluster (Table 4), clear distinct genetic structure (Figure 2) and separated chemical profile (Figure 3), tend to confirm that the cluster corresponds to the subpecies *Lavandula angustifolia* sp. *pyrenaica*. The chemical profile is dominated by linalool, borneol, eucalyptol, linalyl acetate, and cis-sabinene hydrate and appears to be closer to the chemical composition of the Spike lavender *Lavandula latifolia* Medik (eucalyptol/camphor/linalool; [24]) than to the chemical composition of the subspecies *L. angustifolia* sp. *angustifolia* (linalyl acetate/linalool/caryophyllene oxide; [6]). This finding is in accordance with the phylogenetic study in the genus *Lavandula* using plastidic marker [3]. The authors found that *L. angustifolia* sp. *pyrenaica* was genetically more related to *L. latifolia* than *L. angustifolia* sp. *angustifolia* and suggests treating *L. angustifolia* sp. *pyrenaica* as a separate species. Moreover, the occurrence of the two subspecies *L. angustifolia* sp. *angustifolia* and *L. angustifolia* sp. *pyrenaica* is described as mutually exclusive while *L. angustifolia* sp. *pyrenaica* and *L. latifolia* overlap [1], suggesting possible gene flow. Our results provide additional arguments to treat *L. angustifolia* sp. *pyrenaica* as a distinct species. Two plants from Spain were genetically assigned to the Spanish population/species but chemically assigned to the Provencal cluster. We planned to replicate these samples but could not. Unfortunately, all the plant material was used for the first analysis and the samples definitely disappear with the end of the lavender trials at the CNPMAI. Therefore, we cannot conclude with certainty on the status of these two plants.

The Calabrese population, the southernmost population of *Lavandula angustifolia*, constitutes a second genetically homogeneous unit. Chemical and genetic data show an important divergence from all other populations, suggesting the absence of gene flow and local adaptation. The differentiation is even stronger than with *L. angustifolia* sp. *pyrenaica* and the Calabrese population is probably a relict that has been isolated for a long time. Médail and Diadema [25] indicated that the south of Italy was a glacial refugia during Pleistocene climatic cycles which led to isolated populations. According to Passalacqua et al. [2], our results confirm that the Calabrian plants constitute a distinct species from *L. angustifolia* sp. *angustifolia* and *L. angustifolia* sp. *pyrenaica* (*Lavandula austroapennina* N.G.Passal., Tundis, Upson *sp. nov.*).

All individuals in the center of the native range around Provence, including the Italian Alps, belong to the same genetic population and express the typical chemotype of the cultivated lavenders (Figure 3). Indeed, these wild populations were unambiguously assigned to the same chemical cluster than the most cultivated French varieties. The traditional lavender harvest in “baïassières” (areas where wild lavender spontaneously grows) was abandoned after the First World War and the cultivation increased thanks to the possibility of multiplying selected plants from natural populations by cuttings. Our results seem to confirm this domestication scenario with the wild Provencal population that would have yielded the cultivars Maillette, Matheronne, Diva, and Rapido. The genetic and chemical homogeneity, despite the important number of individuals (N = 65), indicate important gene flow within the central area [26]. The possibility of gene flow via pollinators between wild and cultivated plants would also tend to homogenize plants in this area.

Among the largest genetic units including all French plants and the Italian Piedmontese population, a second chemotype was clearly distinct from the traditional chemotype. Constituted by individuals from south of Massif Central (Figure 1), in Lot, Lozere, and Aveyron, this chemotype is characterized by the highest amount of valuable esters lavandulyl and linalyl acetates. The most valuable compounds that should be maximized according to the international standards (linalool, lavandulol, linalyl, and lavandulyl acetate) sum up to 72.7% on average, while it is only 44.2% in cultivated plants. Moreover, the chemical profile of cluster 1 matches the other requirements of the international standards (Table 1), e.g., low proportion of camphor (less than 0.5%) and eucalyptol (less than 1%). Thus, our study highlights an interesting alternative chemotype for the production of high quality essential oil. The production of lavender EO in this region exist since a long time but the production of EO is anecdotal compared to the tonnage of essential oil obtained from traditional varieties. However, the EO produced on this area has a reputation of very high quality [27]. Cluster 1 appears to be promising for future breeding programs to create new cultivars with high quality EO and plants well adapted to low altitude (lowest population from cluster 1 at 250 m high).

The composition in secondary metabolites of the inflorescence of true lavender was highly variable with significant differences for the number of compounds and the total amount of VOCs between clusters (Figure 2). The proportion of monoterpenes and sesquiterpenes was relatively conserved among clusters and was similar to the literature reviewed [28,29]. The intra-specific variability in plant secondary metabolites is common and was discussed in a review from Moore et al. [19]. Quantitative and qualitative variation may play an important role in defining the ecological niche of plants. Several authors show the implications of terpenes in many ecological processes such as pollinator attraction [30], host-parasite interaction [31], or tolerance to abiotic stresses [32,33]. However, the mechanisms of action remain to be discovered and the demonstration of the adaptive advantages of one compound is still a challenge for ecologists. By growing plants from different geographical areas in a common garden, we have reduced the part of chemical variability due to phenotypic plasticity. Our results showed that populations expressed the chemotype associated with their native localities. These four geographically distinct chemotypes showed that terpenes have a role in local adaptation. We found that certain compounds were associated with the southernmost populations: linalool, eucalyptol, β-caryophyllene, borneol, camphor, and cis-sabinene hydrate. An adaptive role of these molecules to warmer and/or drier climate could be hypothesized. To go beyond the correlation, further experimental tests should be conducted to show evidence of causal links. For example, reciprocal transplantation or experimental fumigation of plants exposed to variable temperature conditions could be investigated. In Spike lavender in Spain, Munoz-Bertomeu et al. [13] found that warmer and drier climates favor the accumulation of eucalyptol in plants, while cooler climates favor linalool. In another study on *L. latifolia*, Herraiz-Peñalver et al. [24] found that linalool and camphor contents are inversely correlated and that camphor contents increased at low altitude. These results are not completely in agreement with ours because linalool was positively correlated with eucalyptol (Spearman Rho= 0.48, S = 20619, *p* < 0.001) and camphor (Spearman Rho = 0.53, S = 18831, *p* < 0.001), and associated with southern populations. The enantiomeric analysis of linalool should provide an explanation to these incongruent results. In Bulgarian crops, the major form is R-linalool with a ratio 95/5 [34] but environmental conditions may change this ratio. Terpinen-4-ol was positively correlated with latitude and negatively correlated with altitude. Guitton et al. [6] indicated that terpinen-4-ol became the major compound when lavender was fruiting and constituted an indicator of precocity. Populations originating from low altitude show more terpinen-4-ol because they might flower earlier in the season. Possibly, southern populations transplanted in northern latitude (the growing area is at northern latitude 48.40) may delay their flowering, explaining the lowest amount of terpinen-4-ol in southern populations with latitude < 43.00.

In the context of climate change, pinpointing compounds potentially involved in plant tolerance to high temperature or water stress is of particular interest for a sustainable production. In the case of *Thymus vulgaris*, climate change has already modified the natural repartition of the six chemotypes: freezing sensitive chemotypes (with high contents in carvacrol and thymol) are now present in populations that were exclusively composed of freezing tolerant plants 30 years ago (linalool, thuyanol-4, α-terpineol, and geraniol chemotypes) [35]. These changes in natural populations can be transposed to crops [36]. Climate change induces environmental variations that should be addressed to maintain a sustainable production of lavender essential oil. This involves maintaining the pool of chemical and genetic variability and creating more resilient cultivars. Our study highlights one interesting chemotype (cluster 1, in Massif Central) that grows at lower altitude (down to 250 m) while true lavender usually grows between 500 and 1800 m. The culture of lavender currently extends further and further in the north of France and into the plains. A cultivar that give high quality EO while at low altitude and without experiencing Mediterranean summer drought is thus promising. Remarkable compounds associated with southern latitudes could also be favorable for future breeding programs, even if further analyses are needed to demonstrate the implication of these terpenes in the adaptation to higher temperature or water stress. Very few studies have experimentally demonstrated the action of terpenoids in response to abiotic stress. The use of experimental approaches in controlled conditions needs to be developed in the future to establish causal links and elucidate the exact mechanisms by which terpenes act on abiotic factors and offer adaptive advantages to plants.

## 4. Materials and Methods

### 4.1. Plant Sampling

From 1996 to 2002, an exhaustive census of wild sites of true lavender (*Lavandula angustifolia* Miller) was conducted over its natural range by Bernard Pasquier, director of the French national conservatory of fragrant, medicinal, aromatic, and industrial plants (*Conservatoire National des Plantes à Parfum, Médicinales, Aromatiques et Industrielles, CNPMAI*) and expert botanist of the genus *Lavandula*. Two hundred and eighty-two sites were identified as wild lavender sites (not recently escaped from cultivation). This work allowed us to update the map of natural distribution of true lavender (gray shading in Figure 1, Supplementary Materials Table S1) and to constitute an ex situ bank of wild diversity by collecting seeds from each of the sites. For varietal selection screening, a sample of these sites was selected to be representative of the distribution area in terms of density and location. Seeds were sown in a common garden of CNPMAI at Milly la Forêt (France; lat.: 48.40, long.: 2.48). Plants were grown with similar cultural and environmental conditions. Ten years later, within this pool, we selected 14 sites to be representative of the natural range, without having any knowledge *a priori* on their belonging to a genetic population or a given chemotype [1]. This selection included three sites at the edge of the range (two originating from Spain and one in the south of Italy (Table 2, Figure 1, Figure S1). Five plants of 10 years old were sampled per site during two consecutive days with sunny weather (from 10 a.m. to 4 p.m.). Pre-wilting is traditionally used before extracting EO [37,38] and was applied to our sampling. Ten full-blooming inflorescences were collected per plant and placed in paper envelopes for drying and storage before VOCs extraction. On the same plants, 30 fresh green leaves were collected, dried, and stored in silica gel. For comparison with domesticated lavender, four different cultivars amongst the most cultivated in France (Maillette, Diva, Rapido, and Matheronne) were included in the study. Plants were sampled in five fields located within the main production area in the south of France (with two different fields of Matheronne). Samples were collected with the same protocol, at the same stage at full-blooming on a sunny day.

### 4.2. Genetic Fingerprinting

Total DNA was extracted from 10 mg of dried leaves with the DNeasy 96 extraction kit (QIAGEN) according to the manufacturer’s instructions. The AFLP procedure was carried out with restriction enzymes EcoRI and MseI following the protocol of Nicolè et al. [39]. Three primer combinations were selected for clear profiles, homogeneous distribution of DNA fragments, and repeatability: EcoRI-AGAT/MseICAT, EcoRI-ATCC/MseI-GTC, and EcoRI-ATC/MseI-CAG. AFLP fragments were separated by electrophoresis on an ABI Prism 3100 DNA sequencer (Applied Biosystems, Foster City, California). The electropherograms were analyzed with GeneMapper (Applied Biosystems) and Peak Scanner v1.0 (Applied Biosystems). To produce reliable genetic data, we discarded low-quality DNA samples (i.e., with degraded genomic DNA) and performed negative controls at each step of the procedure. AFLP fragments shorter than 50 bp and monomorphic fragments were discarded. The profiles were normalized and each marker was scored in presence or absence based on the R script described in Herrmann et al. [40]. We used a very restrictive coding method based on 13 duplicated samples to achieve a repeatability of 100%. Eighty-two individual plants were successfully genotyped with 206 highly repeatable polymorphic markers. Results were reported in a binary matrix of presence/absence of each marker for each individual.

### 4.3. VOCs Extraction and Analysis

For each sample, 600 mg of dried inflorescences were placed in 4 mL of hexane containing 200 µL·L^−1^ of cis-3-hexenol as internal standard (Sigma-Aldrich). After an overnight incubation at 4 °C, supernatant was collected and stored at −20 °C. Supernatant was analyzed in gas chromatography-mass spectrometry (GC-MS) with an Agilent 6850N GC, equipped with a 5973N mass selective detector and a DB-5 MS column (30 m × 0.25 mm with a 0.25 µm film-Agilent 122-5532). The initial temperatures of injector and detector were at 250 and 260 °C, respectively. The gas carrier was helium at a flow rate of 1 mL min^−1^. The injection volume was 2 µL with a split ratio of 5:1. The oven’s initial temperature was maintained at 40 °C during 6 min after injection; then ramped at 1 °C min^−1^ to 60 °C, 2 °C min^−1^ to 140 °C, and 12 °C min^−1^ to 240 °C. The oven temperature would finally remain at 240 °C for 5 min. Finally, we could obtain reliable profiles for 90 samples (65 from wild sites and 25 from cultivated fields; see Table 2). The preprocessing of GC-MS analyses was performed with the R package MSeasy [41] that detects putative compounds in a complex metabolic mixture. In brief, all mass spectra of all 90 samples were computed in one matrix. Unsupervised hierarchical clustering was then performed to group similar spectra into one cluster. We visually checked the correspondence of each cluster to a putative molecule and merged or split the cluster when needed. Similarly to AFLP markers, we checked for repeatability of chemical profiles by analyzing five samples thrice. We selected compounds that allow 100% repeatability for the number of peaks and 99.5% repeatability for peak areas. This process eliminated minor peaks whose area was close to the limit of detection. The percentage of total area represented by the repeatable compounds was calculated.

For quantification purposes, we checked the quasi perfect linearity between the range of concentration from 0 to 300 mg dry inflorescence per mL and the areas of peaks (r^2^ = 0.99). A relative area for each compound was thus calculated per dry mass unit and by correcting for differential evaporation during sample handling using the internal standard. A commercial range of n-alkanes C8–C20 (Fluka) was co-injected to calculate retention indices. Identification of compounds was ultimately based on the comparison of retention time and mass spectra to commercial standards when available (lab bank of 80 standards). Identification was otherwise performed by using reference libraries of mass spectra [42] and retention indices. We finally constructed a matrix with individuals in rows and compounds in columns, and relative area of compounds in each cell (internal standard equivalent per g of dry inflorescence).

### 4.4. Statistical Analyses

The data analysis pipeline consisted of four main steps: identifying homogeneous groups of samples using chemical and genetic data separately, pinpointing the compounds that discriminate the chemically homogeneous groups, analyzing the structure of genetic variability, and finally evaluating the concordance between the genetic and chemical structure of the individuals. The first two steps were implemented by using the integrative approach of discriminant analysis of principal components (DAPC, [21]) implemented in the package adegenet [43]. DAPC implemented both unsupervised and supervised classification, and proposed several tools to optimize and evaluate the classification. In the first step, DAPC performed a principal component analysis (PCA). PCA provided new synthetic axes that optimized the representation of the square of the Euclidean distances between individual profiles. This Euclidean distance can be calculated from quantitative data and binary data. The only assumption is that these distances have a biological meaning, which is the case here (chemical and genetic dissimilarity). By definition, these new axes (the principal components) are orthogonal and thus independent from each other. DAPC optimized the number of principal components to be used as new independent variables for unsupervised classification. This step avoided the multicolinearity problems encountered with chemical data (the synthesis and production of one compound is not independent of others [44]). A sequential K-means and model selection with Bayesian information criterion (BIC) infer the optimal number of clusters among the individuals without labeling the data (no information about geographical localization and site membership; *function find.clusters*, [22]). We therefore ran DAPC to define and describe clusters of chemically related individuals and thereafter to identify genetically homogeneous groups.

In a second step, for chemical data, discriminant analysis ran on the optimal number of clusters to identify discriminant compounds among chemotypes. Discriminant analysis requires the construction of an assignment model, associated with conditions of application. Linear discriminant analyses require the number of variables (compounds) to be less than the number of observations (individuals) to avoid overfitting problems. This condition is rarely met with chemical data and fingerprinting genotype. Likewise, abundance of compounds is rarely normally distributed for minor compounds and this prevents us from using parametric methods. However, partial least square discriminant analysis (PLSDA [23]) and discriminant analysis of principal components (DAPC, [22]) overcame these constraints. Both methods were implemented and gave the same structure of samples. Only the results of DAPC are presented.

Similar to the classification procedure of DAPC, the discriminant function of DAPC used the first principal components as the new variables. These new synthetic variables were thus independent, normally distributed, and less numerous than the number of samples, allowing the application of a linear discriminant model. This discriminant model was evaluated by repeated stratified cross-validation (*function xvalDapc*). Redundancy analysis (RDA) was achieved to test for difference of the overall chemical composition between the chemical groups identified by DAPC [23].

Based on the binary matrix of fingerprinting genotypes, analysis of molecular variance (AMOVA) was performed using GenAlex 6.5 [45] to estimate the hierarchical structure of the genetic diversity among genetic clusters identified by DAPC, among sampled sites and within sampled sites. The genetic differentiation between the genetic groups identified with DAPC was determined using PHI_PT_, an analogue of F_st_ adapted to binary data. Significance of pairwise estimates of PHI_PT_ was obtained by using 9999 permutations and confidence interval at 95%, by 10,000 re-samplings.

The fourth step is to evaluate the concordance between the structure of the individuals based on genetic and chemical dataset. A procrustean co-inertia analysis (PCIA) was performed. Contrary to the bidimensional Mantel test, PCIA provides the capacity to deal with higher dimensional spaces. Indeed, PCIA fitted a set of points to another based on the ordination of distance matrices. Euclidean distance was calculated for chemical data and the Jaccard index on AFLP band sharing was used on the genetic dataset. These distance matrices were thus ordinated by principal coordinate analysis (PCoA) and the concordance of the two PCoAs was evaluated by a statistic called m^2^ which varies between 0 (no concordance) and 1 (perfect concordance). The significance of this statistic was tested using the function *protest* (package vegan [46]). We applied the DIABLO framework of the R package mixOmics [47] because it integrated simultaneously the two data sets in a supervised analysis and provided a visual representation of the congruency of the two datasets (DIABLO stands for Data Integration Analysis for Biomarker discovery using Latent variable approaches for Omics studies). The factor was the cluster membership of each sample identified previously by DAPC from chemical data. Finally, non-parametric Spearman correlation tests were implemented on major and most discriminant compounds with latitude and altitude of the 14 native populations. All statistical analyses were performed on R version 3.5.1 (CRAN R project). The significance level was set to *p* = 0.05.

## Figures and Tables

**Table 1:** International standards for the oil of *Lavandula angustifolia* Miller (norm for population lavender, i.e., lavender grown exclusively from seed, spontaneous or cultivated mainly in the south of France).

	NF ISO3515:2004
β-phellandrene	Max 0.5
3-octanone	Max 2
camphor	Max 0.5
limonene	Max 0.5
eucalyptol	Max 1.0
α-terpineol	Max 1.0
terpinene-4-ol	2–6
Cis-ocimene	4–10
Trans-ocimene	1.5–6.0
linalool	25–38
linalyl acetate	25–45
*lavandulol*	Min 0.3
*lavandulyl acetate*	Min 2.0

**Table 2:** Characteristics of the 14 wild lavender sampled sites (site) and the cultivated populations (cult). The characteristics are: number of samples analyzed (N); characteristics of the original localization: Longitude (Long), Latitude (Lat) in decimal degree (DD) and range of altitudes in meter (m); the mean percentage of the total area represented by the 63 repeatable compounds (%Total area), percentage of monoterpenes (%Mono), and percentage of sesquiterpenes (%Sesqui) ± standard deviation; the total amount of extracted compounds (internal standard equivalent per g of dry inflorescence) as well as the total number of compounds ± standard deviation. The codes of sampled sites are: major letter for country (F: France, S: Spain, I: Italy); minor letter for local area (ar: Aragon, lot: Lot, loz: Lozère, av: Aveyron, dr: Drôme, ha: Hautes-Alpes, bdr: Bouche-du-Rhône, am: Alpes-Maritimes, ahp: Alpes-de-Hautes-Provence, pi: Piémont, ca: Calabre), and Cult for cultivated lavender.

Site	N	Long (DD)	Lat (DD)	Altitude (m)	% Total Area	% Mono	% Sesqui	Total Amount	Number of Compounds
Fahp	5	6.6	44.17	1400	83.4 ± 10.9	73.2 ± 14.1	6.9 ± 3	1.4 ± 0.7	29.6 ± 5.2
Fam	3	7.08	43.76	772–800	59.0 ± 18.5	48.4 ± 17	9.0 ± 3.1	0.8 ± 0.3	10.7 ± 2.3
Fav	5	5.45	43.9	450–600	92.0 ± 3.8	78.4 ± 8.5	12.1 ± 6	4.5 ± 4.7	32.0 ± 9.8
Fbdr	3	5.66	43.32	725–800	71.0 ± 4.1	53.3 ± 9.6	17.0 ± 13.2	2.4 ± 1.1	22.3 ± 3.1
Fdr	5	5.09	44.85	590–600	72.4 ± 9.9	65.6 ± 9.4	5.0 ± 2.8	1.3 ± 1	17.6 ± 5.2
Fha1	4	6.69	44.93	1450–1600	77.8 ± 16.4	70.3 ± 18.9	6.0 ± 3	2.3 ± 1.9	21.8 ± 6
Fha2	5	5.94	44.06	755–800	92.7 ± 3.1	78.5 ± 7.1	11.9 ± 3.5	2.3 ± 1.3	29.0 ± 7.7
Fha3	5	5.85	44.12	1635–1800	80.9 ± 7.1	67.7 ± 9.8	10.8 ± 3.6	1.2 ± 0.6	27.2 ± 7.8
Flot	5	1.53	44.95	250–400	88.7 ± 7.3	77.6 ± 9.1	6.8 ± 2.4	7.4 ± 3.5	41.0 ± 7.6
Floz	5	3.37	44.22	980–1000	92.0 ± 7.6	85.8 ± 9	4.1 ± 1	16.0 ± 8.1	39.0 ± 7.4
Ica	5	16.05	39.9	1150–1200	66.7 ± 12.3	58.0 ± 11.9	8.2 ± 1.1	35.2 ± 9.2	55.2 ± 0.8
Ipi	5	7.23	44.34	1275–1400	74.5 ± 10.1	66.3 ± 8.6	4.8 ± 1.6	1.1 ± 0.4	13.4 ± 1.5
Sar1	5	0.21	42.57	1000	88.5 ± 2.4	79.7 ± 4.3	7.5 ± 3	12.8 ± 3.9	48.4 ± 2.9
Sar2	5	−0.55	40.51	1450–1600	84.4 ± 2.7	76.8 ± 3.9	5.1 ± 1.4	5.4 ± 2	43.0 ± 2.5
Cult	25	-	-	-	83.0 ± 9.1	70.0 ± 12.1	10.7 ± 4.8	1.1 ± 0.6	29.1 ± 7.9

**Table 3:** Results of hierarchical AMOVA (analysis of molecular variance) based on the fingerprinting AFLP genotypes of 82 samples of true lavender. The significance level was set to *p* = 0.05.

Source of Variation	df	Sum of Squares	Estimated Variance	Percentage of Variance	PHI-Statistics	*p*
Between genetic clusters	2	440.3	14.83	53	PHI_RT_ = 0.535	<0.001
Among pop within clusters	16	422.3	4.18	15	PHI_PR_ = 0.324	<0.001
Within populations	63	549.9	8.73	31	PHI_PT_ = 0.685	<0.001

**Table 4:** Pairwise PHI_PT_ values between genetic clusters (below diagonal) and probability associated with the permutation test for pairwise differentiation (above diagonal; 999 permutations). The significance level was set to *p* = 0.05.

	French Genetic Cluster	Calabrese Genetic Cluster	Spanish Genetic Cluster
French genetic cluster	-	0.001	0.001
Calabrese genetic cluster	0.557	-	0.001
Spanish genetic cluster	0.552	0.752	-

## Supplementary Materials

The following are available online at https://www.mdpi.com/2223-7747/9/12/1640/s1, Table S1: Table indicating the chemical composition for each population with mean percentage +/-Standard Deviation (SD) for all compounds, percentage of the total represented by identified compounds (% Identified), percentage of monoterpenes (% Monoterpene), percentage of sesquiterpenes (% Sesquiterpene) and the mean number of compounds and total relative area (relative area per dry mass unit and relative to the internal standard). For each compound, retention time (RT); retention indices (RI) which are calculated Kovats indices are shown. Figure S1: Map showing the true lavender sites identified during the CNPMAI surveys from 1996 to 2002 (black dots). The sites used in this study are represented with white symbols.
