# Erratum: Despinasse et al. Structure of the Chemical and Genetic Diversity of the True Lavender over Its Natural Range. *Plants* 2020, *9*, 1640

**DOI:** 10.3390/plants10050922

**Published:** 2021-05-04

**Authors:** Yolande Despinasse, Sandrine Moja, Catherine Soler, Frédéric Jullien, Bernard Pasquier, Jean-Marie Bessière, Sylvie Baudino, Florence Nicolè

**Affiliations:** 1 Université de Lyon, UJM-Saint-Etienne, CNRS, LBVpam FRE 3727, 23 rue du Dr Paul Michelon, F-42023 Saint-Etienne, France; yolande.despinasse@gmail.com (Y.D.); sandrine.moja@univ-st-etienne.fr (S.M.); c_soler@orange.fr (C.S.); jullien@univ-st-etienne.fr (F.J.); sylvie.baudino@univ-st-etienne.fr (S.B.); 2 Laboratoire Cogitamus, 1 ¾ rue Descartes, 75005 Paris, France; 3 Conservatoire National des Plantes Médicinales Aromatiques et Industrielles, Route de Nemours, 91490 Milly La Forêt, France; bernard.pasquier@cnpmai.net; 4 Ecole d’Enseignement Supérieur en Chimie de Montpellier, 8 Rue de l’École Normale, 34090 Montpellier, France; jeanmarie.bessiere@gmail.com

As requested by the Editorial Office, the authors remove the scientific consortium “Camille Nous” from the author list and the Author Contributions section in the published paper [1]. To recognize the consortium‘s contribution, the authors had claimed a double affiliation of two of the authors to the Cogitamus laboratory [2]. The original article has been updated.
