# Supplementary material for: Structure of the Chemical and Genetic Diversity of the True Lavender over Its Natural Range
Source: Plants (Basel). 2020 Nov 24;9(12):1640. doi: 10.3390/plants9121640 (PMC7760402; doi:10.3390/plants9121640)
Supplement: Supplementary file 1 [file plants-09-01640-s001.zip › supplementary-xml/FigureS1.pdf]

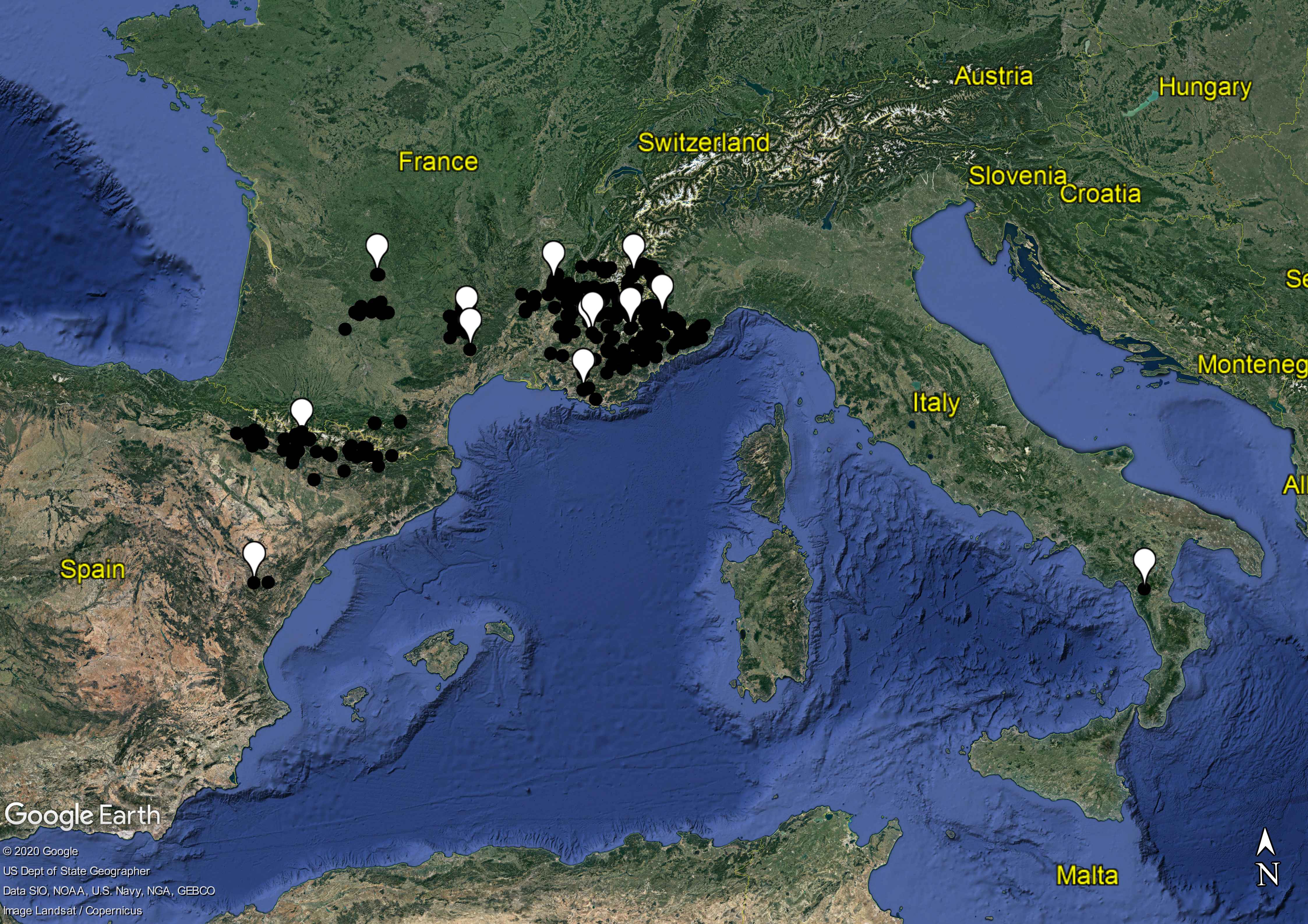
Austria
Hungary
Switzerland
France
Slovenia
Croatia
Se
Monteneg
Italy
All
Spain
Malta
N
Google Earth
© 2020 Google
US Dept of State Geographer
Data SIO, NOAA, U.S. Navy, NGA, GEBCO
Image Landsat / Copernicus
